# Tailoring the ethanol selectivity of SnO_2_-based MEMS gas sensors *via* WO_3_-loading in double-pulse-driven mode

**DOI:** 10.1039/d5ra08502k

**Published:** 2025-12-09

**Authors:** Tao Ren, Koichi Suematsu, Yusuke Shimada, Yusuke Inomata, Tetsuya Kida, Ken Watanabe, Kengo Shimanoe

**Affiliations:** a Interdisciplinary Graduate School of Engineering Sciences, Kyushu University Kasuga Fukuoka 816-8580 Japan; b Department of Advanced Materials Science and Engineering, Faculty of Engineering Sciences, Kyushu University Kasuga Fukuoka 816-8580 Japan suematsu.koichi.682@m.kyushu-u.ac.jp shimanoe.kengo.695@m.kyushu-u.ac.jp; c Faculty of Advanced Science and Technology, Kumamoto University Kumamoto 860-8555 Japan; d Kumamoto University, Institute of Industrial Nanomaterials (IINa) Kumamoto 860-8555 Japan

## Abstract

This work demonstrates a strategy to tailor the ethanol selectivity of SnO_2_-based MEMS gas sensors by combining WO_3_ loading with a pulse-driven heating mode. This dynamic operating mode separates the sensing signal, allowing for the distinction between an initial transient response in the target gas atmosphere (*S*_p_) and a total response (*S*_i_). WO_3_-loaded SnO_2_ nanoparticles (NPs) were synthesized *via* hydrothermal treatment and impregnation methods. The optimal 1.5 mol% WO_3_-loaded SnO_2_ (1.5–W–Sn) exhibited highly dispersed WO_3_ species and an enhanced specific surface area. At measurement temperature of 350 °C, the 1.5–W–Sn sensor showed selectivity toward 10 ppm ethanol with an *S*_p_ value significantly higher than those for other VOCs. *In situ* diffuse reflectance infrared Fourier transform spectroscopy (DRIFTS) measurements confirmed that the 1.5–W–Sn sample enhanced ethanol adsorption at low-temperature. More critically, results of the temperature programmed reaction after ethanol adsorption confirmed that WO_3_ loading altered the ethanol reaction pathway, which was evidenced by ethylene desorption. This altered pathway, coupled with the rapid combustion of its byproducts during the measurement step is responsible for the high *S*_p_ value. The combination of pre-adsorption and an altered reaction pathway, enables the sensor's marked selectivity to ethanol. These findings underscore a powerful design strategy, where material properties and dynamic operation modes are tailored the selective detection.

## Abstract 2

A highly selective ethanol sensor was achieved using WO_3_-loaded SnO_2_ under pulse heating mode. The strategy relies on pre-adsorption of ethanol at low temperatures, followed by rapid reaction during the heating pulse to generate a transient signal.

## Graphical abstract

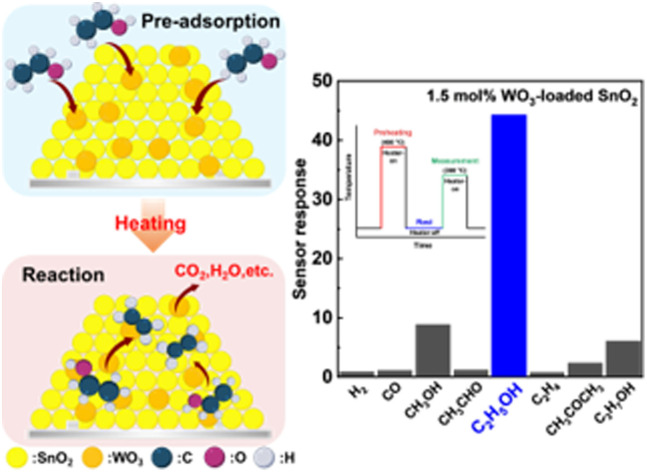

## Introduction

The detection of volatile organic compounds (VOCs) is of paramount importance in fields such as environmental monitoring, industrial safety, and public health.^1–5^ In this context, chemiresistive gas sensors based on metal oxide semiconductors (MOS) have emerged as one of the most promising technology platforms, owing to their advantages of high sensitivity, fast response, and excellent stability. Among them, tin dioxide (SnO_2_), a representative n-type semiconductor, has demonstrated notable performance in detecting reducing gases.^6–8^ However, conventional MOS gas sensors face the limitations of large size and high power consumption, which limit their application in portable and Internet of Things (IoT) devices. The advent of Micro-Electro-Mechanical Systems (MEMS) technology, which integrates microheaters and sensing elements onto a silicon substrate, has drastically reduced the device size and power consumption, providing the sensors with a rapid thermal response.^9,10^ This capability has transformed sensor operation, turning the microheater from a passive heater into an active modulator of gas-sensing physicochemical processes. This has invigorated research along two key pathways: the development of advanced sensor driving strategies and the material designs.

In the realm of sensor driving strategies, researchers have exploited the capability of MEMS for rapid temperature control to develop dynamic temperature modulation techniques.^11–13^ Basic strategies, such as the pulse-driven mode, can effectively amplify the sensing signal and enhance sensitivity by dividing the sensing process into discrete temperature stages.^14^ Building upon this concept, a more advanced double-pulse-driven (DP) mode has been developed. This mode separates the sensing process into three steps. First, a high-temperature preheating step removes the adsorbates and increases the amount of adsorbed oxygen ions (O^2−^) on the surface of the material. In the following rest (heater-off) step, the target gas molecules are adsorbed onto the surface and condensed in the sensing layer. Next, during the measurement step, these adsorbates are combusted rapidly upon heating, which generates a highly amplified signal. Consequently, this approach allows the signal amplification to reach its theoretical maximum sensitivity, as exemplified by the detection of toluene at the parts-per-trillion (ppt) level.^15^ Nevertheless, the poor selectivity of pristine SnO_2_ remains a deep-rooted issue. Relying solely on the optimization of the operation mode provides only limited improvement in selectivity when discriminating between gas molecules with similar functional groups or the same carbon number. This constitutes a core challenge in the development of high-performance sensors.

The receptor function plays a key role in the material design of high-performance gas sensors, as its sensitization effect mainly stems from chemical interactions between the sensing material and gas molecules, such as redox, acid–base, and catalytic properties.^8,16–18^ In our previous work on thick-film sensors, we confirmed that loading the acidic receptor WO_3_ on the SnO_2_ surface enhances selectivity for ethanol.^19^ This result was attributed to the acid–base and catalytic properties, which alter the ethanol reaction pathway from dehydrogenation to dehydration under high-temperature range, isothermal heating condition. However, under the isothermal heating mode, the selectivity is largely dominated by the surface reaction, resulting in only a limited enhancement, typically a 2.3-fold and 3.2-fold difference compared with acetone and acetaldehyde, respectively.^19^ This limitation suggests that strictly focusing on steady-state surface reactions is insufficient for further enhancing selectivity. The origin of selectivity is likely governed not only by the steady-state reaction, but also by processes that take place under non-equilibrium conditions, such as the adsorption/desorption dynamic properties of ethanol compared with interfering gases. Therefore, the pulse-driven mode provides an ideal platform for investigating whether selective detection of low concentration ethanol can be achieved through the pre-adsorption and subsequent reaction processes of the WO_3_-loaded SnO_2_ surface.

In this work, we introduced WO_3_ as a receptor on SnO_2_ surface and employed the double-pulse-driven mode to investigate its effect on selectivity. We demonstrated that WO_3_ enhances selectivity for alcohol detection by leveraging both its surface acidity and its influence on the reaction pathway, thereby achieving notably high ethanol sensing performance. This study thus validates an effective design strategy in which by a receptor with tailored surface properties is combined with an operating mode that amplifies its effects, offering a clear approach toward the development of highly selective sensors for sensitive applications such as medical diagnostics.

## Experimental

### Materials synthesis

SnO_2_ nanoparticles were synthesized *via* hydrothermal method followed by calcination. A 1.0 M SnCl_4_·5H_2_O (98%, Fujifilm Wako Pure Chemical Corporation, Japan) solution was dropwise added under stirring to a 1.0 M NH_4_HCO_3_ (99.0%, first grade, Fujifilm Wako Pure Chemical Corporation, Japan) solution, resulting in the formation of a white stannic acid gel. After centrifugation to remove Cl ions, the gel was dispersed into deionized water, and the pH was adjusted to 10.5 using ammonia solution (28%, Fujifilm Wako Pure Chemical Corporation, Japan). The resulting suspension was transferred to a stainless reaction vessel with a PTFE inner cylinder and subjected to hydrothermal treatment at 200 °C for 10 h. The obtained translucent sol was distilled at 60 °C and dried overnight. The obtained powder was ball-milled for 5 h and subsequently calcined at 600 °C for 3 h under an oxygen flow to prepare SnO_2_ nanoparticles. WO_3_-loaded SnO_2_ nanoparticles were prepared *via* an impregnation method followed by calcination. First, WO_3_ precursor was synthesized by an acidification process. A 0.5 M (50 mL) aqueous solution of sodium tungstate dihydrate (Na_2_WO_4_·2H_2_O, 99.0–100.5%, Kishida Chemical Co., Ltd, Japan) was added dropwise to 3.15 M (300 mL, pH of −0.8) H_2_SO_4_ (95%, Fujifilm Wako Pure Chemical Corporation, Japan) under continuous stirring, leading to the formation of a yellow gel of crystalline WO_3_·*n*H_2_O. The gel was aged at 30 °C for 24 h and subsequently washed several times with distilled water by centrifugation until the pH of the supernatant reached 5–6 to remove residual Na ions. The obtained gel was dried at 80 °C to yield a yellow powder, which was then mixed with deionized water to obtain the WO_3_ precursor. Then, 10 mL of this precursor solution, containing the stoichiometric amount of WO_3_ for 1, 1.5, and 3 mol% relative to SnO_2_, was added dropwise to a suspension of SnO_2_ powder (1 g) in deionized water (20 mL). The mixture was stirred for 24 h, centrifuged, and dried, and the resulting powder was calcined at 500 °C under an O_2_ flow for 2 h to obtain WO_3_-loaded SnO_2_ NPs. Hereafter, the neat SnO_2_ and the 1, 1.5, and 3 mol% WO_3_-loaded SnO_2_ samples will be referred to as SnO_2_, 1–W–Sn, 1.5–W–Sn, and 3–W–Sn, respectively.

### Characterization

The crystal structures of as-synthesized SnO_2_ and WO_3_-loaded SnO_2_ materials were analyzed by X-ray diffraction (XRD; MiniFlex, Rigaku, Japan) using Cu Kα radiation (*λ* = 0.15418 nm) at 40 kV and 30 mA over a 2*θ* range of 20–80° with a scanning rate of 2°·min^−1^. The loading amount of WO_3_ in WO_3_-loaded SnO_2_ materials was evaluated by wavelength-dispersive X-ray fluorescence spectroscopy (WDX, Rigaku, ZSX Primus II, Japan). The microstructures and elemental distributions of as-prepared materials were examined by field-emission scanning electron microscopy (FE-SEM; JSM-IT800SHL, JEOL, Japan) equipped with an energy-dispersive X-ray (EDX) analyzer. STEM and EDX analyses were carried out on a scanning transmission electron microscope (JEM-ARM200F, JEOL, Japan) operated at an accelerating voltage of 200 kV, which was equipped with a silicon drift detector for EDX measurements. The pore size distribution and specific surface area were determined from N_2_ adsorption–desorption isotherms measured on a BELSORP-mini II analyzer (Microtrac Bell, Japan), and calculated by Barrett–Joyner–Halenda (BJH) method and Brunauer–Emmett–Teller (BET) method, respectively.

*In situ* diffuse reflectance infrared Fourier transform spectroscopy (DRIFTS) measurements were performed on a Nicolet iS50 FTIR spectrometer (Thermo Fisher Scientific, USA) equipped with a mercury–cadmium-telluride (MCT) detector with a spectral resolution of 4 cm^−1^. The sample powder (∼20 mg) was placed in a ceramic sample holder within a reaction chamber fitted with BaF_2_ windows. The chamber was connected to a gas flow system, maintaining a constant total flow rate of 100 mL min^−1^. First, the sample was pre-treated at 450 °C for 1 h in a flow of synthetic air to clean the surface. Subsequently, the sample was cooled to 50 °C and held for 30 min in synthetic air to stabilize, after which a background spectrum was recorded. The gas atmosphere was then switched to 50 ppm ethanol in synthetic air for 30 minutes. Finally, the temperature was raised to 350 °C, and the entire procedure of *in situ* DRIFTS measurement was repeated.

Temperature-Programmed Reaction (TPR) measurements were used to analyze the gas adsorption and reaction characteristics on the material surfaces. The experimental setup included a gas mixing system, a reaction chamber, and a quadrupole mass spectrometer (QMS; PrismaPlus QMG220, PFEIFFER, Germany). First, the sample powders (SnO_2_ and WO_3_-loaded SnO_2_) were prepared by being pressed at 0.23 tonf cm^−2^ for 5 minutes and then ground into 250–710 µm agglomerates. For the ethanol-TPR procedure, 100 mg of the prepared sample was placed in the reaction chamber. The calculated space velocity was approximately 90 000 h^−1^. The sample was initially preheated at 450 °C for 1 hour in a 21% O_2_/Ar flow of 100 mL min^−1^. After preheating, a 100 ppm ethanol/21%O_2_/Ar mixture was introduced into the reaction chamber at 50 °C for 2 hours. The gas flow was then switched back to 21% O_2_/Ar. In the final step, the samples were heated to 450 °C at a rate of 5 °C min^−1^ under flowing 21% O_2_/Ar. The emission of ethanol and its oxidation products was monitored by a QMS detector. The analysis focused on the signals for the following mass-to-charge ratios (*m*/*z*): 44 (mainly assigned to CO_2_ and partially other byproducts of ethanol combustion), 29 (mainly assigned to CH_3_CHO and partially C_2_H_5_OH), and 27 (mainly assigned to C_2_H_4_ and partially CH_3_CHO and C_2_H_5_OH).

### Gas sensor fabrication and measurements

SnO_2_ and WO_3_-loaded SnO_2_ nanoparticles were each mixed with glycerin at a mass ratio of 2 : 1 to prepare the corresponding pastes, which were deposited onto microsensor device as the sensing layers using a capillary-assisted micromanipulation system, as described previously.^20^ The obtained devices were then dried under vacuum at 180 °C for 2 h and subsequently calcined in flowing synthetic air at 480 °C for 2 h using integrated microheaters. The morphology and thickness of the sensing layers on the microsensors were examined using a 3D laser scanning confocal microscope (VK-X1000, KEYENCE, Japan).

The electrical resistance of the MEMS-type sensors was evaluated using a double-pulse-driven mode in an apparatus consisting of a gas mixing system, a quartz-type sensing chamber, and an operating system. During measurements, the microheater was controlled to maintain a preheating temperature of 450 °C by applying a voltage of 1.9 V, while the measurement temperatures were set to 350, 300, and 250 °C by applying voltages of 1.5, 1.3, and 1.1 V, respectively. The preheating and measurement periods were 10 s each, while the rest (heater-off) time was set to 30 s. This rest time was optimized based on the results shown in Fig. S5. The electrical resistance was recorded every 0.01 s. During each measurement cycle, the resistance was defined as *R*_a_ (electrical resistance in the synthetic air atmosphere), *R*_g,i_ (at the beginning of the measurement step in the target gas), and *R*_g,e_ (at the end of the measurement step in the target gas), and three sensor response parameters were defined as *S*_e_ (*R*_a_/*R*_g,e_), *S*_p_ (*R*_g,e_/*R*_g,i_), and *S*_i_ (*R*_a_/*R*_g,i_). Schematic of heater voltage and temperature profiles during double-pulse driven operation and the definition of the sensor response is shown in Fig. S1. Standard gases of the target analytes, including H_2_, CO, methanol, ethanol, acetaldehyde, ethylene, acetone, and propanol, each with an initial concentration of approximately 125 ppm in N_2_, were diluted in N_2_ and mixed with pure O_2_ and N_2_ under dry conditions to prepare test gases containing 21% O_2_/N_2_. All sensing measurements were repeated for at least 10 consecutive cycles for each gas concentration and temperature condition. The sensor response values reported in the figures were derived from the final stable cycle of these measurements. The reproducibility of the sensor response was verified by calculating the relative standard deviation (RSD) based on consecutive stable cycles, ensuring the validity of the presented data.

## Results and discussion

### Materials characteristics

**Fig. 1:**
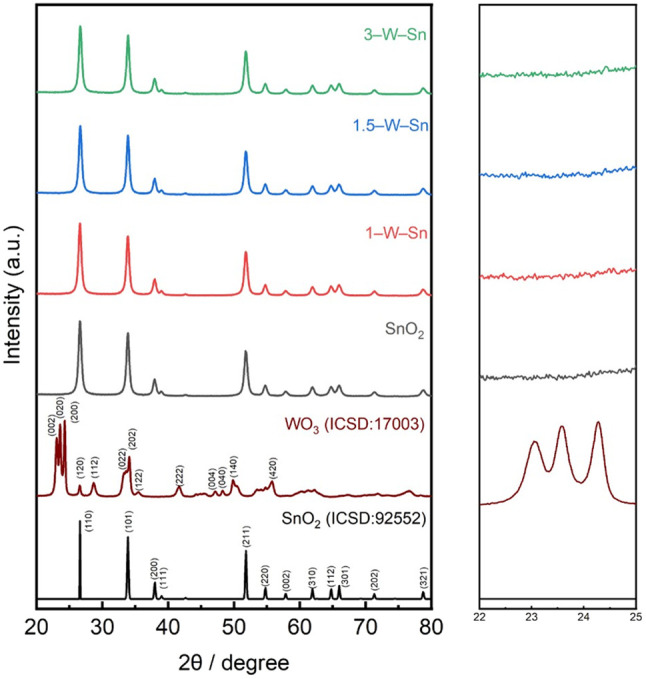
XRD patterns of SnO_2_, 1–W–Sn, 1.5–W–Sn, and 3–W–Sn samples.

The actual content of WO_3_ in the WO_3_-loaded SnO_2_ materials was evaluated by WDX analysis based on the calibration using the mixture of WO_3_ and SnO_2_ materials. For the samples prepared with nominal WO_3_ concentrations of 1, 1.5, and 3 mol%, the measured contents were found to be 0.81, 1.24, and 2.35 mol%, respectively. Fig. 1 displays the X-ray diffraction (XRD) patterns of the neat SnO_2_ and the WO_3_-loaded SnO_2_ samples. All patterns exhibited identical diffraction peaks, which matched well with the tetragonal rutile structure of SnO_2_ (space group: *P*4_2_/*mnm*, ICSD: 92552), confirming that all as-synthesized materials possessed the same SnO_2_ crystal phase. Furthermore, the average crystallite sizes were calculated using the Scherrer formula from the (110), (101), and (211) planes. The sizes for neat SnO_2_, 1–W–Sn, 1.5–W–Sn, and 3–W–Sn were determined to be approximately 16 nm. These results confirm the nanostructure of the powders and indicate that the crystallite size of SnO_2_ remained largely unaffected by the WO_3_ loading. Notably, no characteristic diffraction peaks for WO_3_ were observed in any samples. The absence of the main diffraction peaks for monoclinic WO_3_, such as the (002), (020), and (200) planes typically found in the 2*θ* range of 22–25°, is likely attributed to the high dispersion or the small loading amount of WO_3_ particles on the surface of the SnO_2_ nanoparticles. Although the low loading amount precluded the detection of specific diffraction peaks, our previous XPS results on identically prepared samples confirmed the presence of W^6+^ species, indicating that the loaded tungsten exists as highly dispersed crystalline WO_3_.^19^

**Fig. 2:**
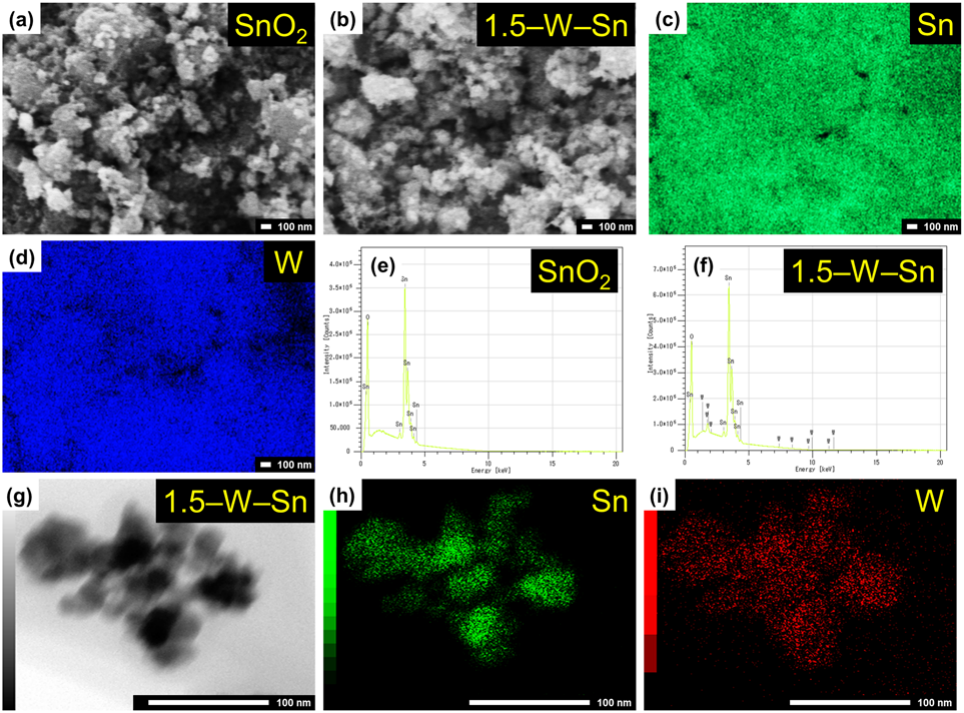
Morphological and compositional analysis of the samples. (a and b) FESEM images of SnO_2_ and 1.5–W–Sn; (c and d) corresponding EDX elemental mappings of Sn and W for the 1.5–W–Sn sample; (e and f) EDX spectra of SnO_2_ and 1.5–W–Sn, respectively; (g) TEM image of 1.5–W–Sn sample, and (h and i) its corresponding EDX elemental mappings of Sn and W.

Fig. 2a and b show FE-SEM images of neat SnO_2_ and 1.5–W–Sn. The corresponding images for the 1–W–Sn and 3–W–Sn are shown in Fig. S2. The SEM image of neat SnO_2_ reveals a porous structure composed of finely aggregated nanoparticles. The presence of visible pores among the aggregates plays a crucial role in promoting gas diffusion into the sensing layer. In the case of 1.5–W–Sn, the morphology is highly similar to that of the neat SnO_2_, maintaining the same fine particle size and loosely packed, porous structure. Notably, no discrete and large particles corresponding to WO_3_ are observed.

The elemental composition and spatial distribution were further verified by EDX analysis. The elemental mapping images for Sn (Fig. 2c) and W (Fig. 2d) reveal a uniform distribution of both elements. This confirms that the tungsten species are finely and homogeneously dispersed throughout the composite. Complementing this spatial analysis, a comparison of the EDX spectra was used to confirm the elemental composition. The spectrum of the neat SnO_2_ (Fig. 2e) shows only the characteristic peaks for Sn and O, confirming the purity of the base material. In contrast, the spectrum of the 1.5–W–Sn sample (Fig. 2f) clearly displays additional peaks corresponding to the tungsten Mα emission at 1.774 keV, demonstrating the successful loading of tungsten. The TEM image and corresponding EDX maps of the 1.5–W–Sn sample (Fig. 2g–i) confirmed the high dispersion of the WO_3_. As seen in the elemental maps for Sn (Fig. 2h) and W (Fig. 2i), the tungsten species are uniformly spread over the SnO_2_ surface. This result is in agreement with the XRD data and underscores the importance of such a well-dispersed WO_3_.

**Fig. 3:**
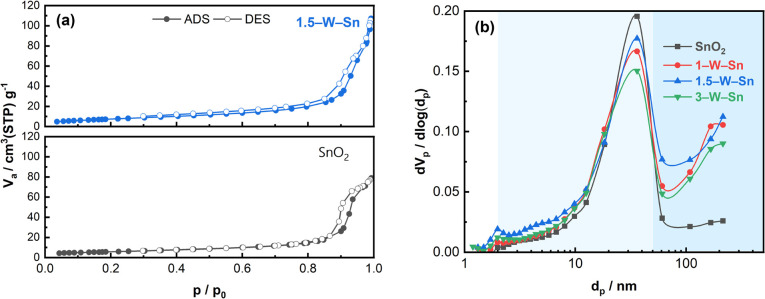
(a) N_2_ adsorption–desorption isotherms and (b) BJH pore size distribution of SnO_2_ and WO_3_-loaded SnO_2_ NPs.

The N_2_ adsorption–desorption isotherms and corresponding BJH pore size distributions for neat SnO_2_ and WO_3_-loaded SnO_2_ nanoparticles are illustrated in Fig. 3. The N_2_ adsorption–desorption isotherms for 1–W–Sn and 3–W–Sn are shown in Fig. S3. As shown in Fig. 3a, both samples exhibit type IV isotherms with a distinct hysteresis loop, characteristic of mesoporous materials. At a relative pressure (*p*/*p*_0_) close to 1.0, the WO_3_-loaded SnO_2_ samples reach a higher adsorbed volume compared to the neat SnO_2_, indicating the presence of macropores formed by interparticle aggregation. Since the total adsorption volume is directly related to the material's porosity, this indicates that the introduction of WO_3_ effectively increases the total pore volume and specific surface area of the material. The pore size distribution using the BJH method is illustrated in Fig. 3b. The specific surface areas are 20.5, 24.7, 28.5, and 26.4 m^2^ g^−1^ for neat SnO_2_, 1–W–Sn, 1.5–W–Sn, and 3–W–Sn, respectively. Correspondingly, their pore volumes are 0.117, 0.149, 0.163, and 0.138 cm^3^ g^−1^. Notably, the specific surface area and pore volume are observed to be larger than previously reports, which may be attributed to the ball-milling process. This enhancement occurs because ball-milling concurrently reduces large secondary particles to increase surface area and promotes their reassembly into loose agglomerates. For the neat SnO_2_, the peak pore diameter is centered at approximately 35 nm. After the introduction of WO_3_, the main pore diameter for the composite samples shows a slight decrease to around 30 nm. Meanwhile, WO_3_ loading increases the volume of larger pores with diameters over 50 nm, which may facilitate the effective penetration of gas molecules into the gas sensing layer.^21^ However, considering Knudsen diffusion, such pore structure modifications are not expected to significantly affect the overall sensing performance under the pulse-driven mode, which is instead primarily governed by surface properties. Overall, the 1.5–W–Sn composite exhibits the most developed porous structure with the highest specific surface area and largest pore volume, suggesting enhanced gas adsorption capabilities.

### Sensing properties

**Fig. 4:**
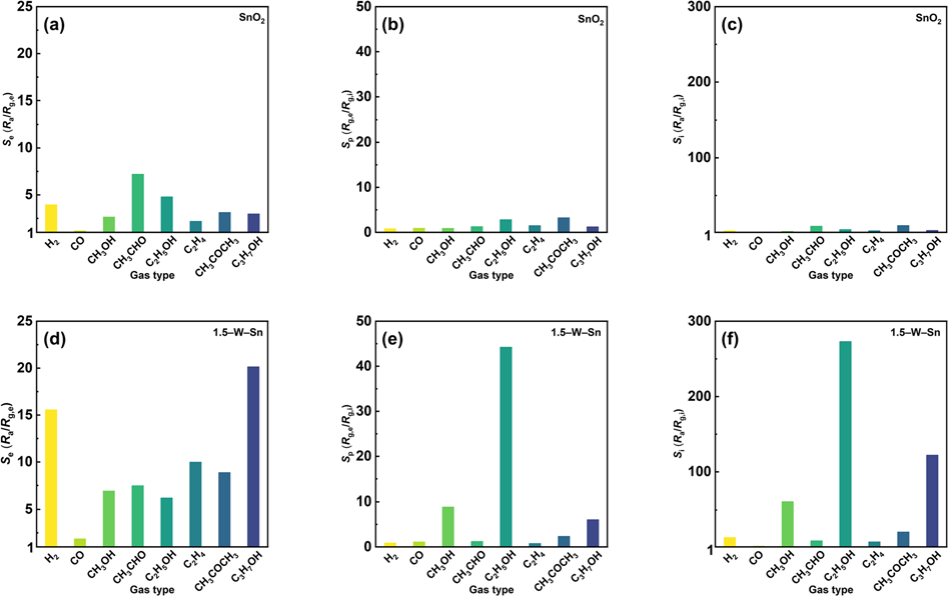
The sensor responses (*S*_e_, *S*_p_ and *S*_i_) of sensors containing (a–c) SnO_2_ and (d–f) 1.5–W–Sn to 10 ppm various target gases at measurement temperature of 350 °C.

First, the physical morphology and film thickness of the fabricated MEMS sensor devices are shown in Fig. S4. The results confirm that all sensors exhibit a uniform and consistent surface morphology, and the thickness of the sensing layer was well controlled within the range of 25–30 µm for all devices, ensuring a reliable comparison of sensing performance. Gas sensing performance was evaluated using a range of representative analytes, including typical gases such as H_2_ and CO, and a series of simple VOCs spanning different functional groups, including alcohols (CH_3_OH, C_2_H_5_OH, C_3_H_7_OH), aldehydes (CH_3_CHO), ketones (CH_3_COCH_3_), and alkenes (C_2_H_4_). The selectivity of the fabricated MEMS sensors was systematically evaluated against 10 ppm of these gases, as shown in Fig. 4. In addition, selectivity data for the neat SnO_2_ and 1.5–W–Sn sensors at 300 and 250 °C are provided in the SI (Fig. S6 and S7). First, the selectivity of the sensor was assessed by the *S*_e_ value (*R*_a_/*R*_g,e_). This parameter reflects the sensor response at the steady-state phase of the heating pulse, which is comparable to the sensor response under isothermal conditions. As shown in Fig. 4a, the neat SnO_2_ sensor exhibited low and non-selective responses to all target gases. For the 1.5–W–Sn sensor (Fig. 4d), while an overall enhancement in response to most gases was observed, it similarly failed to demonstrate the selectivity toward a specific gas. This phenomenon can be understood by considering the properties of the double-pulse-driven mode, which includes adsorption and combustion followed by a steady-state phase. The *S*_e_ value, however, is determined only at the final point of this steady-state period. As demonstrated by its massive transient *S*_p_ value in Fig. 4e, the 1.5–W–Sn sensor possesses an extremely high combustion activity. This intense catalytic activity means that during the steady-state phase (where *S*_e_ is measured), the surface reaction rate is rapid that it becomes limited by the gas diffusion of gas molecules into the sensing layer.^22^ This result clearly indicates that the Se value is unsuitable for evaluating the selective detection capabilities of the 1.5–W–Sn material under this operating mode.

In contrast to the *S*_e_ value, the *S*_p_ value (*R*_g,e_/*R*_g,i_), which represents the transient response originating from the initial rapid combustion, reveals the enhanced performance of the 1.5–W–Sn sensor. The total response *S*_i_ (*R*_a_/*R*_g,i_) then combines these effects. As shown in Fig. 4b, the neat SnO_2_ sensor exhibited negligible *S*_p_ value to all tested gases. However, the 1.5–W–Sn sensor (Fig. 4e) demonstrates a dramatic enhancement in *S*_p_ value, particularly for alcohols. A distinct response toward methanol (*S*_p_ = 8.9), ethanol (*S*_p_ = 44.3), and propanol (*S*_p_ = 6.1) is observed, while other gases show minimal *S*_p_ value. This behaviour is directly related to the sensing mechanism of the double-pulse-driven mode. The *S*_p_ represents the initial, rapid combustion of species that are pre-adsorbed and condensed into the sensing layer during the low-temperature rest step. Here, the higher *S*_p_ values for alcohols indicate that the 1.5–W–Sn surface possesses a strong and preferential adsorption capability for molecules containing hydroxyl groups. However, the higher *S*_p_ value for ethanol compared to methanol and propanol suggests an additional mechanism beyond the gas adsorption affinity. This difference can be attributed to a change in the reaction pathway, which is promoted by loading the acidic WO_3_. At the high temperature of the measurement pulse, these acidic sites are responsible for the dehydration of the ethanol to ethylene.^23,24^ Critically, the *S*_e_ value shown in Fig. 4d indicates that the 1.5–W–Sn sensor exhibits a markedly higher response to C_2_H_4_ than neat SnO_2_. Therefore, the higher *S*_p_ for ethanol likely originates from a two-step reaction process involving ethanol dehydration to ethylene followed by rapid oxidation of the byproducts, resulting in a large transient response. The absence of this reaction pathway for methanol and propanol explains the pronounced selectivity of ethanol. Moreover, this rapid combustion combined with the Se value, determines the total *S*_i_ value (*S*_i_ = *S*_e_ × *S*_p_). As shown in Fig. 4f, the selectivity toward ethanol is further amplified in this value. The 1.5–W–Sn sensor exhibits the most pronounced Si to ethanol (*S*_i_ = 273). This response is 13-fold higher than that of acetone (*S*_i_ = 21) and 30-fold higher than that of acetaldehyde (*S*_i_ = 9.2), demonstrating its excellent selectivity among the tested gases. Therefore, by utilizing the double-pulse-driven mode to leverage both the enhanced adsorption properties and the change in reaction pathway, the 1.5–W–Sn material successfully achieves selective detection of ethanol.

To further verify the sensor performance, the dynamic response curves of the neat SnO_2_ and 1.5–W–Sn sensors to various concentrations of ethanol were recorded at 350, 300, and 250 °C, as shown in Fig. S8. For a detailed investigation of the processes occurring within a single cycle, the data from the final measurement cycle of 10 ppm ethanol at 350 °C was selected. The representative electrical resistance profiles for this single cycle are presented and discussed below. Fig. S9 shows the electric resistance curves in synthetic air and 10 ppm ethanol/21%O_2_/N_2_ during a pulse heating step at 350, 300 and 250 °C under double-pulse-driven mode using (a–c) SnO_2_, (d–f) 1.5–W–Sn, respectively. In the target gas atmosphere, the sensors show dramatically different behaviours, particularly at the beginning of the heating pulse. As shown in Fig. S9a–c, the neat SnO_2_ sensor exhibits only a slight decrease in *R*_g,i_ at the beginning of the heating pulse, quickly reaching a steady-state value (*R*_g,e_) thereafter. In contrast, the 1.5–W–Sn sensor shows a markedly different behaviour, exhibiting a profoundly low initial resistance (*R*_g,i_) at the beginning of the pulse as shown in Fig. S9d–f. This phenomenon can be attributed to its material properties, the 1.5–W–Sn sample possesses the highest specific surface area and largest pore volume. This enhanced porous structure provides a greater number of adsorption sites, allowing a significantly larger amount of ethanol molecules to accumulate. Consequently, when the heating pulse is performed, this large, pre-concentrated load of ethanol is instantaneously combusted, leading to the observed low *R*_g,i_. Following this initial transient combustion, the electrical resistance of the 1.5–W–Sn sensor gradually increases before stabilizing at the steady-state value (*R*_g,e_). This behaviour arises because the pre-adsorbed ethanol molecules have been consumed, and the subsequent reaction becomes limited by the diffusion of ethanol from the atmosphere. Under these conditions, the oxygen species re-adsorb on the surface, capturing electrons and thereby increasing the resistance.

**Fig. 5:**
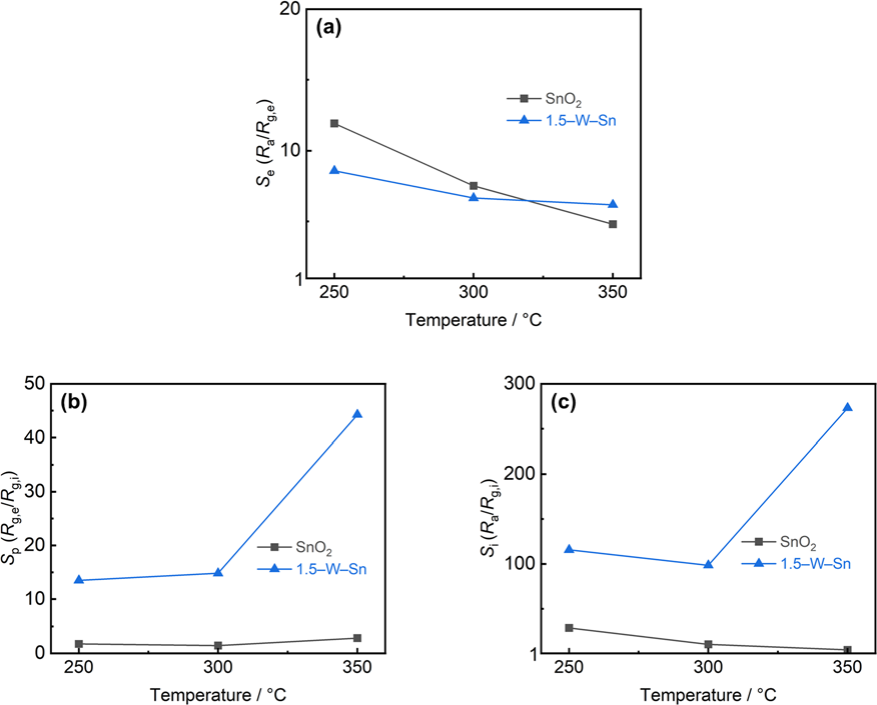
Temperature dependence of (a) *S*_e_, (b) *S*_p_ and (c) *S*_i_ of SnO_2_ and 1.5–W–Sn to 10 ppm ethanol.

The temperature dependence of the sensor performance for both neat SnO_2_ and 1.5–W–Sn was examined, and the results for *S*_e_, *S*_p_, and *S*_i_ toward 10 ppm ethanol are summarized in Fig. 5. For the Se value shown in Fig. 5a, both sensors exhibited a similar trend which the response increased as the operating temperature decreased. The 1.5–W–Sn sensor exhibited a slightly lower Se than neat SnO_2_ at 250 and 300 °C, but surpassed it at a higher temperature of 350 °C. The enhancement observed at 350 °C is particularly significant, as it is consistent with our previous findings in which a change in the reaction pathway enabled selective ethanol detection.^19^

In contrast, the *S*_p_ (Fig. 5b) and *S*_i_ value (Fig. 5c) clearly demonstrate the pronounced influence of WO_3_ loading under the double-pulse-mode. Across all tested temperatures, the 1.5–W–Sn sensor displayed significantly higher *S*_p_ value and *S*_i_ value than the negligible response of neat SnO_2_. Crucially, the *S*_p_ value increased with temperature, reaching a maximum at 350 °C. Consequently, the total response *S*_i_ value (the mathematical product of *S*_p_ and the *S*_e_) also peaked at this temperature. These results indicate that the combined effect of enhanced adsorption and an altered reaction pathway is primarily responsible for the highly selective ethanol detection. Furthermore, the reproducibility of the sensor response was evaluated. As shown in Fig. S10, the 1.5–W–Sn sensor exhibited excellent stability over consecutive measurement cycles toward 10 ppm ethanol at 350 °C. The calculated RSD for *S*_p_ and *S*_i_ were 7.5% and 6.9%, respectively, confirming the stability of the high selectivity obtained in this dynamic operating mode.

### *In situ* DRIFTS and ethanol-TPR results

**Fig. 6:**
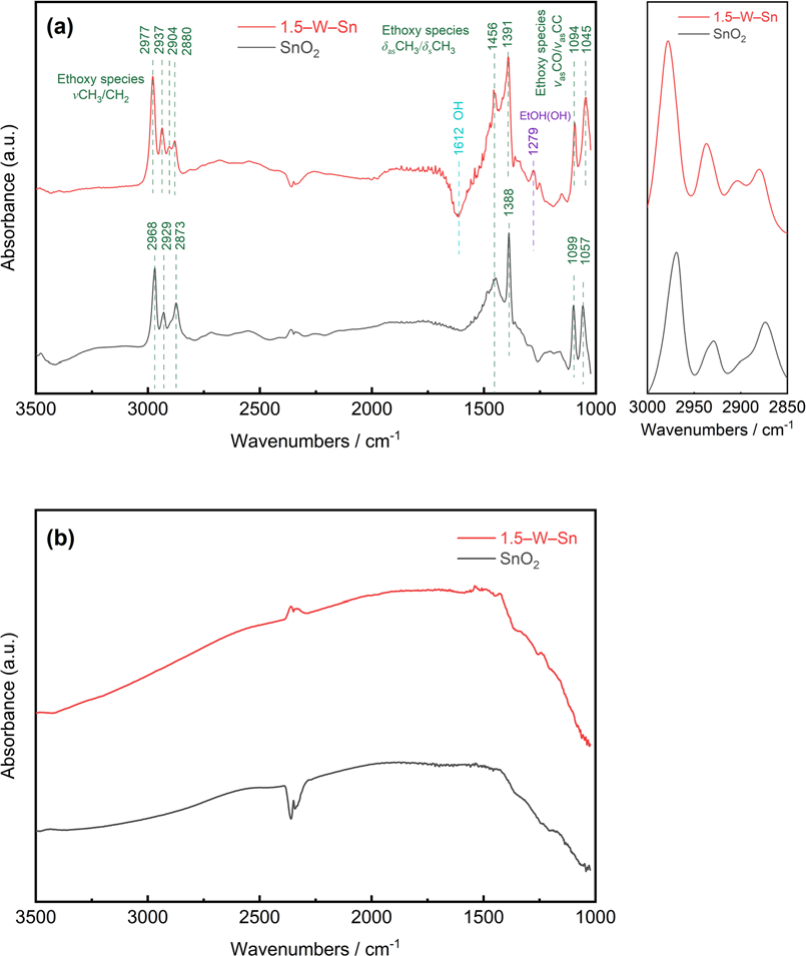
DRIFT spectra during 50 ppm ethanol exposure of SnO_2_ and 1.5–W–Sn NPs at (a) 50 °C and (b) 350 °C in dry condition.

To elucidate the surface processes of ethanol adsorption and reaction, *in situ* diffuse reflectance infrared Fourier transform spectroscopy (DRIFTS) measurements were performed. Fig. 6a displays the spectra of neat SnO_2_ and 1.5–W–Sn samples after exposure to 50 ppm ethanol at 50 °C. This condition was selected to reflect the adsorption behaviour during the rest step of the gas sensor under pulse-driven mode. It should be noted that the absorption bands observed in the 2200–2400 cm^−1^ range are assigned to residual atmospheric CO_2_, rather than a reaction product, and thus do not affect the analysis of the primary surface species. For the 1.5–W–Sn sample, the introduction of ethanol led to the appearance of distinct absorption bands characteristic of surface ethoxy species. Specifically, the C–H stretching vibrations (*ν*(CH_3_/CH_2_)) at 2977, 2937, and 2880 cm^−1^, C–H bending vibrations (*δ*(CH_3_/CH_2_)) at 1456 and 1391 cm^−1^, and C–O stretching vibrations (*ν*(C–O)) at 1045 and 1094 cm^−1^.^25–27^ While the neat SnO_2_ sample exhibited similar characteristic absorption features, the peak positions were found to be slightly shifted compared to those of the 1.5–W–Sn sample. This shift in vibrational frequencies can be attributed to the surface acidity of WO_3_.^28^ In addition, a peak at 1279 cm^−1^ corresponding to the O–H bending mode of molecular ethanol, indicated that a portion of the ethanol is also physically adsorbed.^25^

However, a critical comparison of the two spectra reveals significant differences in surface reactivity. A minor but distinct band at 2904 cm^−1^ is clearly visible on the 1.5–W–Sn sample. This band is primarily assigned to surface ethoxide species,^27^ and its emergence, along with the overall intensification of ethoxy-related bands, indicates a significantly higher accumulation of chemisorbed ethanol species on the 1.5–W–Sn surface compared to neat SnO_2_. Furthermore, previous studies have also suggested that bands in this region can be associated with acyl species,^26^ implying that the WO_3_-loaded surface may not only enhance the accumulation of adsorption species but also promote the initial activation of ethanol. Moreover, the more pronounced negative band around 1612 cm^−1^ observed on 1.5–W–Sn may be attributed to a greater consumption of surface hydroxyl groups,^29,30^ possibly resulting from competitive adsorption, in which ethanol molecules are adsorbed and replace surface hydroxyls to form ethoxy species. While direct quantitative comparison is complex, the fact that all bands related to adsorbed species are substantially more pronounced on the 1.5–W–Sn sample. Taken together, these features indicate that the 1.5–W–Sn material possesses a significantly higher capacity for both the adsorption and initial chemical activation of ethanol molecules.

As the temperature was increased to 350 °C as shown in Fig. 6b, a condition corresponding to the measurement pulse, the spectra changed dramatically. The sharp absorption bands of all adsorbed species completely disappeared from both samples, confirming that all adsorbed species have undergone complete oxidation. The remaining broad bands in the 1200–1700 cm^−1^ region are likely attributable to stable surface byproducts, such as carbonates.^31,32^ This high-temperature behaviour is crucial for the double-pulse driven mode measurement. The combined DRIFTS results provide a comprehensive model for the enhanced sensing performance. During the rest time, the 1.5–W–Sn surface demonstrates enhanced adsorption capability and reactivity, enabling it to accumulate a significantly larger number of ethanol-derived species. During measurement phase, this larger amount of adsorbate is rapidly and completely combusted. This enhanced capacity for low-temperature accumulation and subsequent high-temperature combustion is fundamental to the more pronounced change in electrical resistance, thus resulting in the higher *S*_p_ value observed for the 1.5–W–Sn sensor.

**Fig. 7:**
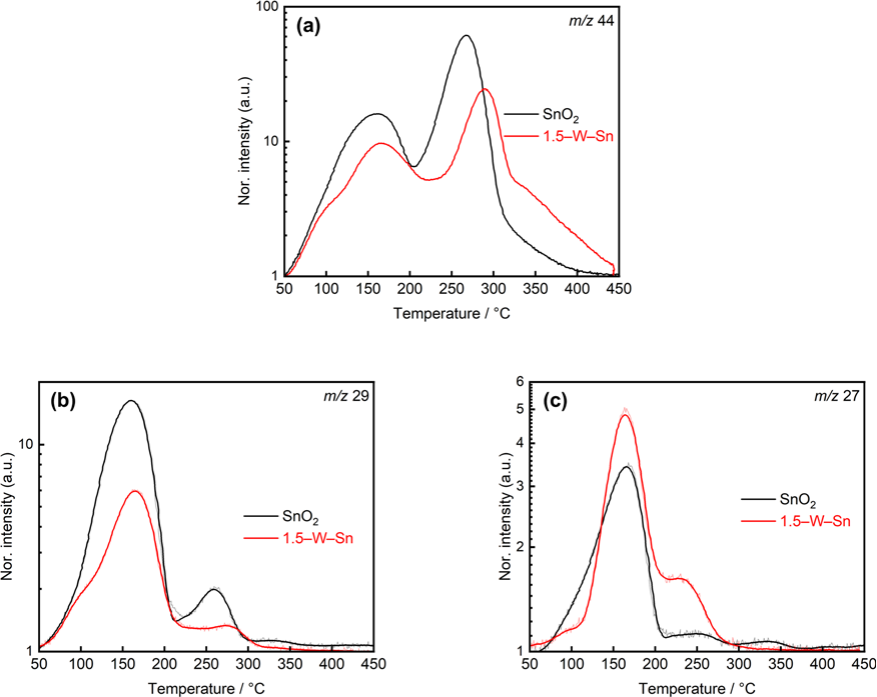
TPR spectra of SnO_2_ and 1.5–W–Sn samples under 21% O_2_/Ar after exposing 100 ppm ethanol during heating procedure from 50 °C to 450 °C at mass-to-charge ratio of (a) 44, (b) 29, and (c) 27.

The temperature-programmed reaction (TPR) results of ethanol for SnO_2_ and 1.5–W–Sn sample were conducted as shown in Fig. 7. The neat SnO_2_ exhibits only one peak (*m*/*z* 27, Fig. 7c) assigned to acetaldehyde, and two distinct peaks (*m*/*z* 29, Fig. 7b) at 160 °C and 260 °C, which may be attributed to the primary desorption of acetaldehyde and the desorption of re-adsorbed byproducts, respectively. The peak at *m*/*z* 44 at 265 °C as shown in Fig. 7a, appearing at a similar temperature to the acetaldehyde peak, indicates the further oxidation of these intermediates to CO_2_.

In contrast, the 1.5–W–Sn sample displays a significantly altered reaction profile. The introduction of WO_3_ clearly separated the reaction pathways of adsorbed gas species by temperature. The profiles of *m*/*z* 44, 29, and 27 all exhibit their initial peaks at 165 °C, corresponding to low-temperature desorption primarily attributed to ethanol dehydrogenation, leading to the desorption of acetaldehyde. Subsequently, a broad peak at a higher temperature of around 230 °C distinct from that of neat SnO_2_ becomes evident in Fig. 7c. This indicates the activation of the dehydration reaction, as confirmed by the pronounced *m*/*z* 27 peak, which mainly corresponds to the ethylene desorption. Correspondingly, the *m*/*z* 44 profile for the 1.5–W–Sn sample in Fig. 7a exhibits two major peaks, followed by a broad shoulder that gradually decreases from 320 °C. The second peak appearing at approximately 290 °C and characteristic of CO_2_ desorption, being significantly shifted to a higher temperature compared with the peak of 265 °C observed for neat SnO_2_.

Furthermore, the broad shoulder observed from 320 °C is likely attributed to the combustion of ethylene, which is generated from ethanol dehydration reaction. This indicates that the complete oxidation of ethylene intermediates requires higher thermal energy compared to the dehydrogenation pathway on neat SnO_2_. However, this elevated activation barrier does not hinder the sensing speed under the DP mode. Since the measurement temperature was set to 350 °C, which is well above the main reaction temperatures observed in TPR results, the thermal energy provided during the heating pulse is sufficient to instantly overcome this barrier. Consequently, the accumulated ethanol species undergo rapid and effectively simultaneous combustion upon the rapid heating, resulting in the high *S*_p_ observed.

This TPR analysis provides strong evidence supporting the proposed sensing mechanism. The higher *S*_p_ value for ethanol observed in the sensitivity can be rationalized by a two-step reaction process. The TPR data in Fig. 7c confirms that the 1.5–W–Sn sample promotes the dehydration reaction of ethanol to ethylene, activating a pathway that occurs minimally on neat SnO_2_. Given that the 1.5–W–Sn sensor exhibits a strong Se response to ethylene, this efficient dehydration pathway explains the excellent *S*_p_ value for ethanol. Furthermore, the shift in the desorption peak temperature of the *m*/*z* 44 in Fig. 7a indicates differences in the adsorption strength of intermediate species. As shown in Fig. S8a–c, the lower *R*_g,i_ might suggested that most acetaldehyde generated through ethanol dehydrogenation desorbs rapidly from the surface without further oxidation, while the subsequent oxidation of a small amount of strongly adsorbed acetate species make a negligible contribution to the *S*_p_ value. In contrast, ethylene likely exhibits stronger adsorption and undergoes oxidation at higher temperatures, thereby playing a dominant role in the enhanced *S*_p_ observed for ethanol. Therefore, the enhanced acidic catalytic property of the WO_3_-loaded surface is identified as the key factor responsible for the selective ethanol detection.

From a theoretical perspective, the loading of WO_3_ on SnO_2_ creates an n–n heterojunction at the interface. Although this electronic interaction can modulate the carrier concentration, the distinctive ethanol selectivity observed in the DP mode cannot be attributed solely to these electronic effects. Instead, the mechanism is primarily governed by surface kinetics and reaction barriers representing chemical sensitization. Theoretical studies have established that acidic oxides effectively lower the activation energy for the dehydration of alcohols.^24^ In contrast, the amphoteric SnO_2_ surface favors the dehydrogenation pathway. This theoretical distinction aligns perfectly with our TPR observations and confirms that the WO_3_ loading provides a kinetically favorable pathway for ethylene generation. This specific pathway triggers the higher *S*_p_ value during the heating pulse.

## Conclusions

In this study, we demonstrated a strategy to enhance the selectivity of SnO_2_-based MEMS gas sensors by loading the acidic receptor WO_3_ under a double-pulse-driven (DP) mode. The optimized 1.5–W–Sn composite, featuring highly dispersed WO_3_ species and an increased surface area, achieved a pronounced *S*_p_ value of 44.3 to 10 ppm ethanol. This resulted in a *S*_i_ value approximately 30-fold higher than acetaldehyde, and significantly greater than those of other interfering gases. *In situ* DRIFTS revealed enhanced ethanol adsorption during the low-temperature rest step. Ethanol-TPR results confirmed the desorption of ethylene, indicating a change in reaction pathway promoted by the loading of WO_3_. The followed combustion of this ethylene byproduct during the heating pulse produces the large and selective transient response. This work establishes a generalizable approach in which material design and dynamic operation are synergistically optimized to control surface reactions, offering a promising direction for developing selective gas sensors. Future work will investigate the effect of humidity on sensor performance to validate its practical applicability.

## Author contributions

Tao Ren: writing – original draft, methodology, investigation, conceptualization, formal analysis, data curation. Koichi Suematsu: writing – review & editing, methodology, funding acquisition. Yusuke Shimada: investigation. Yusuke Inomata: formal analysis. Tetsuya Kida: validation. Ken Watanabe: methodology. Kengo Shimanoe: supervision, funding acquisition.

## Conflicts of interest

There are no conflicts to declare.

## Supplementary Material

RA-015-D5RA08502K-s001

## Data Availability

All data supporting this manuscript are available within the manuscript and the supplementary information (SI). Supplementary information is available. See DOI: https://doi.org/10.1039/d5ra08502k.

## References

[cit1] Wang L., Cheng Y., Gopalan S., Luo F., Amreen K., Singh R. K., Goel S., Lin Z., Naidu R. (2023). Review and perspective: gas separation and discrimination technologies for current gas sensors in environmental applications. ACS Sens..

[cit2] Jian Y., Hu W., Zhao Z., Cheng P., Haick H., Yao M., Wu W. (2020). Gas sensors based on chemi-resistive hybrid functional nanomaterials. Nano-Micro Lett..

[cit3] Khatib M., Haick H. (2022). Sensors for volatile organic compounds. ACS Nano.

[cit4] Paknahad M., Mcintosh C., Hoorfar M. (2019). Selective detection of volatile organic compounds in microfluidic gas detectors based on “like dissolves like”. Sci. Rep..

[cit5] Rosario W., Singh P. K., Tiwari A., Jain U., Avasthi D. K., Chauhan N. (2024). Nanomaterial-based VOC sensing applications and a deep dive into their developmental trends. J. Mater. Chem. A.

[cit6] Das S., Jayaraman V. (2014). SnO_2_: A comprehensive review on structures and gas sensors. Prog. Mater. Sci..

[cit7] Masuda Y. (2022). Recent advances in SnO_2_ nanostructure based gas sensors. Sens. Actuators, B.

[cit8] Yamazoe N. (2005). Toward innovations of gas sensor technology. Sens. Actuators, B.

[cit9] Ruiz A. M., Illa X., Díaz R., Romano-Rodríguez A., Morante J. R. (2006). Analyses of the ammonia response of integrated gas sensors working in pulsed mode. Sens. Actuators, B.

[cit10] Mei H., Zhang F., Zhou T., Zhang T. (2024). Pulse-Driven MEMS NO_2_ Sensors Based on Hierarchical In_2_O_3_ Nanostructures for Sensitive and Ultra-Low Power Detection. Sensors.

[cit11] Suematsu K., Shin Y., Ma N., Oyama T., Sasaki M., Yuasa M., Kida T., Shimanoe K. (2015). Pulse-driven micro gas sensor fitted with clustered Pd/SnO_2_ nanoparticles. Anal. Chem..

[cit12] Tanaka Y. (2024). Recent advancements in physical and chemical MEMS sensors. Analyst.

[cit13] Ji H., Yuan Z., Zhu H., Qin W., Wang H., Meng F. (2022). Dynamic temperature modulation measurement of VOC gases based on SnO_2_ gas sensor. IEEE Sens. J..

[cit14] Suematsu K., Harano W., Oyama T., Shin Y., Watanabe K., Shimanoe K. (2018). Pulse-driven semiconductor gas sensors toward ppt level toluene detection. Anal. Chem..

[cit15] Suematsu K., Harano W., Yamasaki S., Watanabe K., Shimanoe K. (2020). One-trillionth level toluene detection using a dual-designed semiconductor gas sensor: Material and sensor-driven designs. ACS Appl. Electron. Mater..

[cit16] Korotcenkov G., Ivanov M., Blinov I., Stetter J. R. (2007). Kinetics of indium oxide-based thin film gas sensor response: The role of “redox” and adsorption/desorption processes in gas sensing effects. Thin Solid Films.

[cit17] Yamazoe N., Shimanoe K. (2011). Basic approach to the transducer function of
oxide semiconductor gas sensors. Sens. Actuators, B.

[cit18] Jinkawa T., Sakai G., Tamaki J., Miura N., Yamazoe N. (2000). Relationship between ethanol gas sensitivity and surface catalytic property of tin oxide sensors modified with acidic or basic oxides. J. Mol. Catal. A: Chem..

[cit19] Ren T., Suematsu K., Shimada Y., Watanabe K., Shimanoe K. (2025). Surface Modification of SnO_2_ Gas Sensors with WO_3_ for Temperature-Dependent Selective Detection of Ethanol and Acetone. ACS Appl. Electron. Mater..

[cit20] Suematsu K., Oyama T., Mizukami W., Hiroyama Y., Watanabe K., Shimanoe K. (2020). Selective detection of toluene using pulse-driven SnO_2_ micro gas sensors. ACS Appl. Electron. Mater..

[cit21] Suematsu K., Shin Y., Hua Z., Yoshida K., Yuasa M., Kida T., Shimanoe K. (2014). Nanoparticle cluster gas sensor: controlled clustering of SnO_2_ nanoparticles for highly sensitive toluene detection. ACS Appl. Mater. Interfaces.

[cit22] Matsunaga N., Sakai G., Shimanoe K., Yamazoe N. (2002). Diffusion equation-based study of thin film semiconductor gas sensor-response transient. Sens. Actuators, B.

[cit23] Ouayloul L., El Doukkali M., Jiao M., Dumeignil F., Agirrezabal-Telleria I. (2023). New mechanistic insights into the role of water in the dehydration of ethanol into ethylene over ZSM-5 catalysts at low temperature. Green Chem..

[cit24] Kim S., Robichaud D. J., Beckham G. T., Paton R. S., Nimlos M. R. (2015). Ethanol dehydration in HZSM-5 studied by density functional theory: evidence for a concerted process. J. Phys. Chem. A.

[cit25] Schmitt E. A., Krott M., Epifani M., Suematsu K., Weimar U., Barsan N. (2024). Volatile organic compound sensing with WO_3_-based gas sensors: Surface chemistry basics. J. Phys. Chem. C.

[cit26] Ochoa J. V., Trevisanut C., Millet J. M. M., Busca G., Cavani F. (2013). In situ DRIFTS-MS study of the anaerobic oxidation of ethanol over spinel mixed oxides. J. Phys. Chem. C.

[cit27] Rintramee K., Föttinger K., Rupprechter G., Wittayakun J. (2012). Ethanol adsorption and oxidation on bimetallic catalysts containing platinum and base metal oxide supported on MCM-41. Appl. Catal., B.

[cit28] Travert A., Vimont A., Sahibed-Dine A., Daturi M., Lavalley J. C. (2006). Use of pyridine CH (D) vibrations for the study of Lewis acidity of metal oxides. Appl. Catal., A.

[cit29] Huang Y., Kou S., Zhang X., Wang L., Lu P., Zhang D. (2020). Facile fabrication of Z-scheme Bi_2_WO_6_/WO_3_ composites for efficient photodegradation of bisphenol A with peroxymonosulfate activation. Nanomaterials.

[cit30] Bielański A., Lubańska A. (2004). FTIR investigation on Wells–Dawson and Keggin type heteropolyacids: dehydration and ethanol sorption. J. Mol. Catal. A: Chem..

[cit31] Wijnja H., Schulthess C. P. (2001). Carbonate adsorption mechanism on goethite studied with ATR–FTIR, DRIFT, and proton coadsorption measurements. Soil Sci. Soc. Am. J..

[cit32] Singh J., White R. L. (2019). A variable temperature
infrared spectroscopy study of CaA zeolite dehydration and carbonate formation. Spectrochim. Acta, Part A.

