# Supplementary material for: Tailoring the ethanol selectivity of SnO2-based MEMS gas sensors via WO3-loading in double-pulse-driven mode
Source: RSC Adv. 2025 Dec 9;15(57):48913–22. doi: 10.1039/d5ra08502k (PMC12687727; doi:10.1039/d5ra08502k)
Supplement: RA-015-D5RA08502K-s001 [file RA-015-D5RA08502K-s001.pdf]

Supplementary Information (SI) for RSC Advances.
This journal is © The Royal Society of Chemistry 2025

Supporting Information

Tailoring the Ethanol Selectivity of SnO_2-based MEMS Gas Sensors via WO_3-loading in Double-Pulse-Driven Mode

Tao Ren, [a] Koichi Suematsu,*[b] Yusuke Shimada, [b] Yusuke Inomata, [c] Tetsuya Kida, [d] Ken Watanabe, [b] and Kengo Shimanoe*[b]

[a] *Interdisciplinary Graduate School of Engineering Sciences, Kyushu University, Kasuga, Fukuoka 816-8580, Japan.*

[b] *Department of Advanced Materials Science and Engineering, Faculty of Engineering Sciences, Kyushu University, Kasuga, Fukuoka 816-8580, Japan.*

[c] *Faculty of Advanced Science and Technology, Kumamoto University, Kumamoto, 860-8555, Japan.*

[d] *Kumamoto University, Institute of Industrial Nanomaterials (IINa), Kumamoto, 860-8555, Japan.*

Corresponding author e-mail address: suematsu.koichi.682@m.kyushu-u.ac.jp, shimanoe.kengo.695@m.kyushu-u.ac.jp

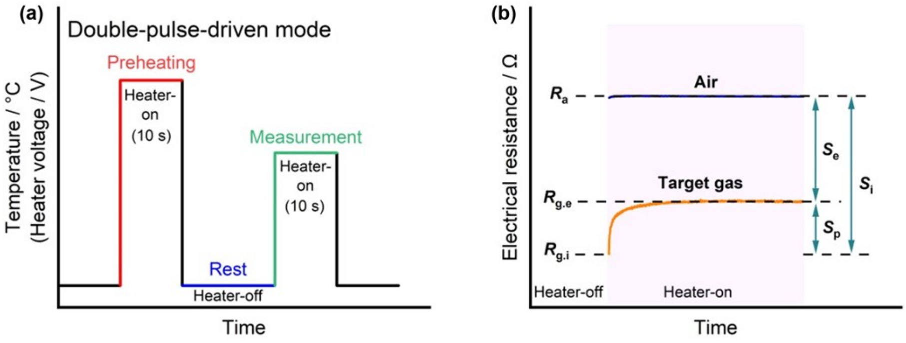

Fig. S1. (a) schematic of heater voltage and temperature profiles during double-pulse driven operation. (b) Definition of the sensor response.

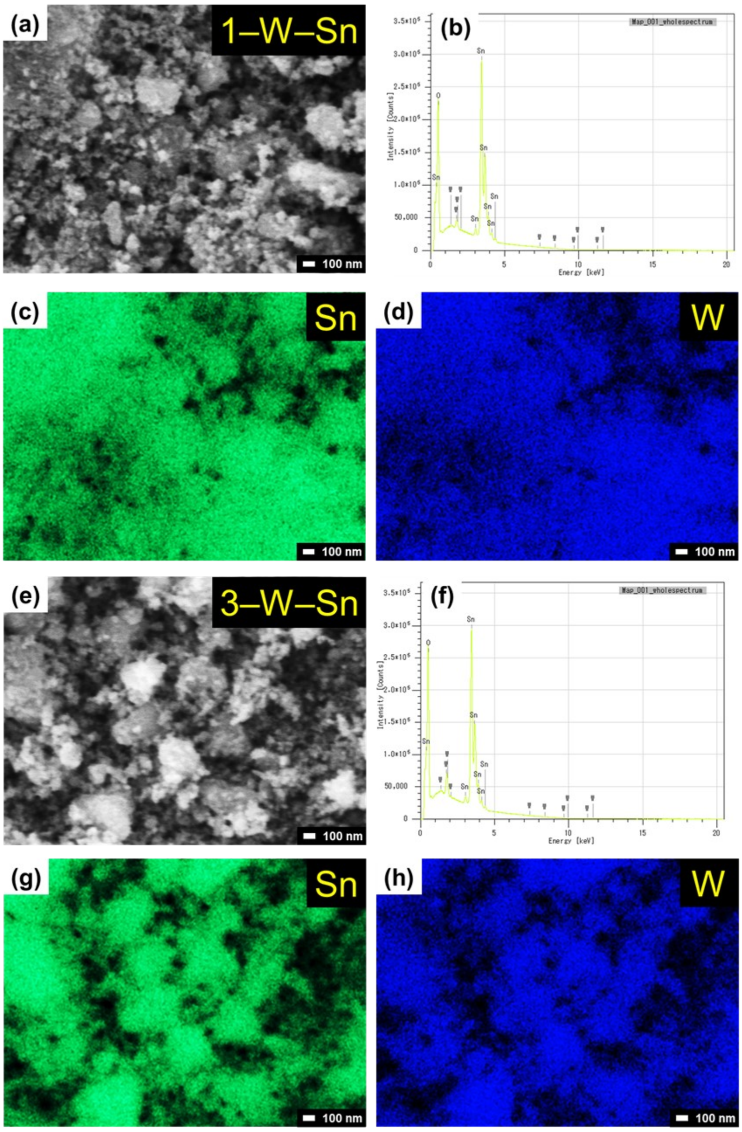

Fig. S2. Morphological and compositional analysis of (a–d) 1–W–Sn, and (e–h) 3–W–Sn sample.

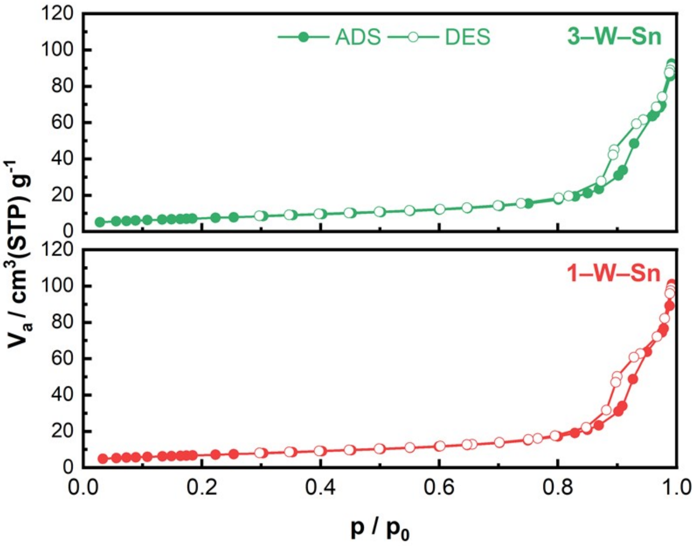

Fig. S3. N_2 adsorption-desorption isotherms of 1–W–Sn and 3–W–Sn sample.

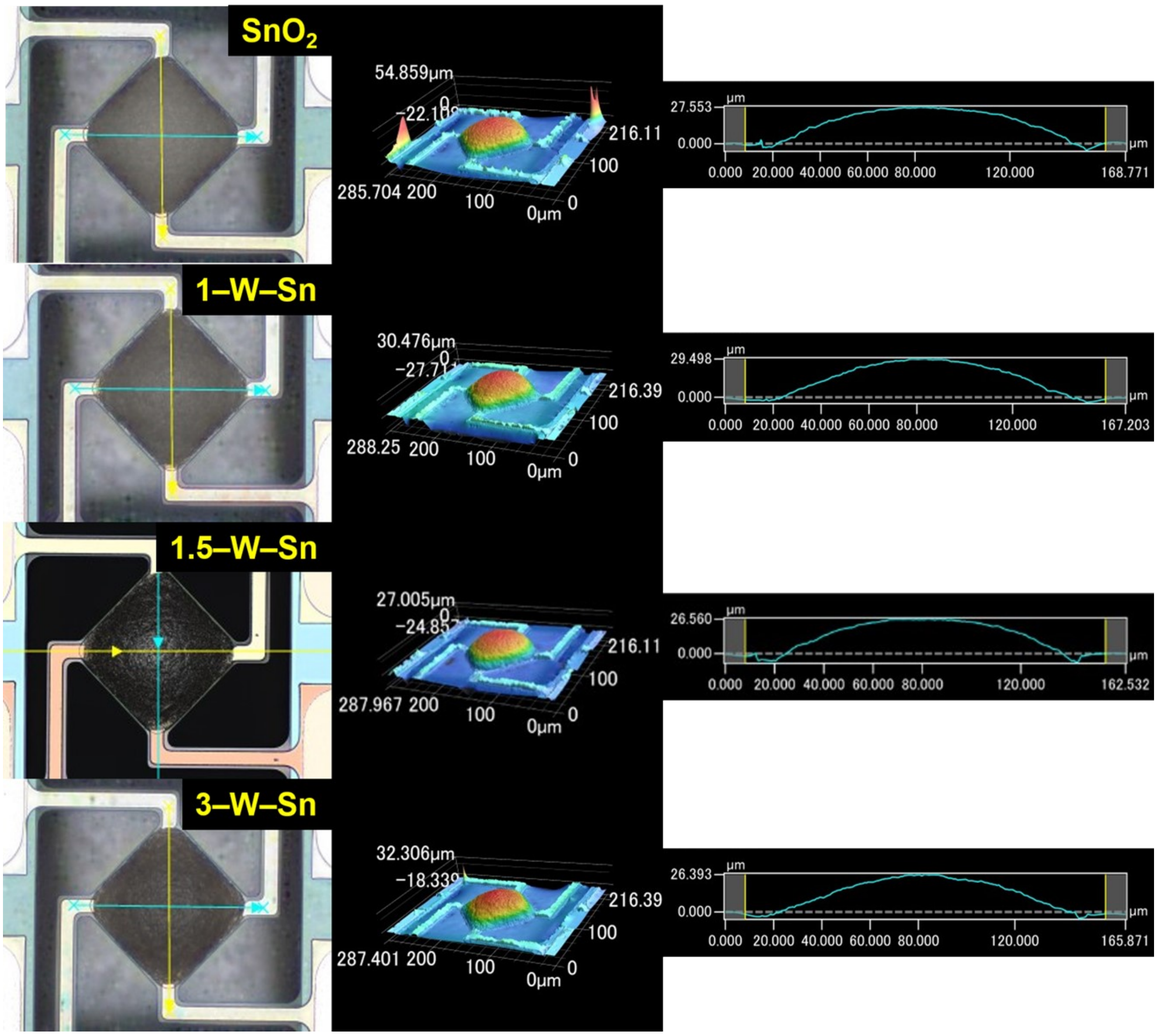

Fig. S4. The physical morphology and film thickness of the fabricated MEMS sensor devices.

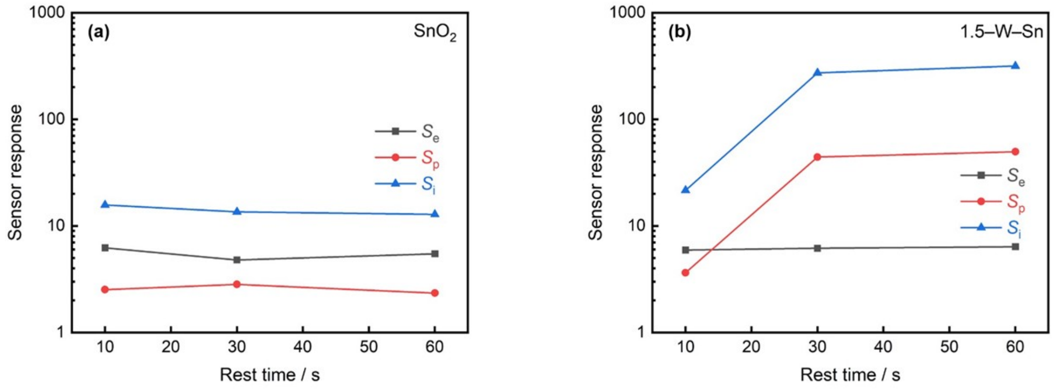

Fig. S5. Rest time dependence of the sensor response for the (a) SnO_2 and (b) 1.5–W–Sn sensors to 10 ppm ethanol at measurement temperature of 350 °C.

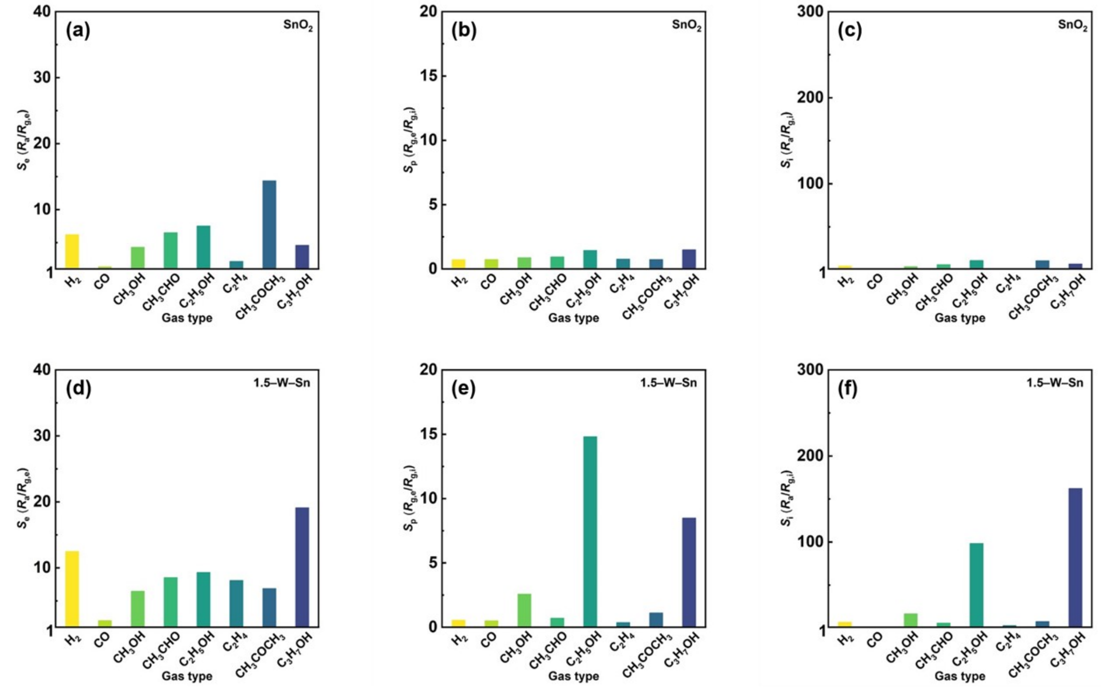

Fig. S6. The sensor responses (S_e, S_p and S_i) of fabricated MEMS sensors containing (a–c) SnO_2, and (d–f) 1.5–W–Sn to10 ppm various target gases at measurement temperature of 300 °C.

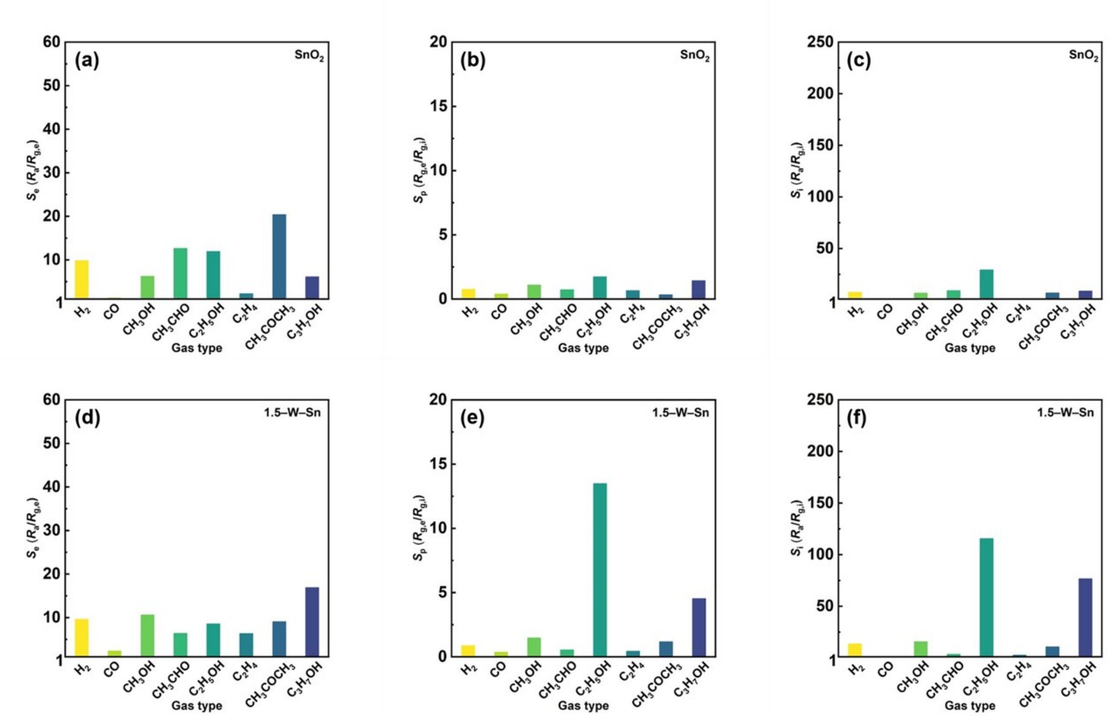

Fig. S7. The sensor responses (S_e, S_p and S_i) of sensors containing (a–c) SnO_2, and (d–f) 1.5–W–Sn to10 ppm various target gases at measurement temperature of 250 °C.

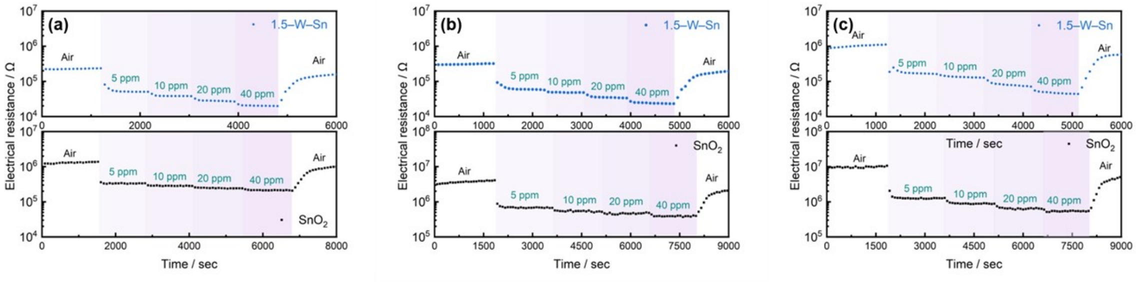

Fig. S8. Dynamic response curves of sensors containing neat SnO_2 and 1.5–W–Sn to ethanol at (a) 350 °C, (b) 300 °C and (c) 250 °C.

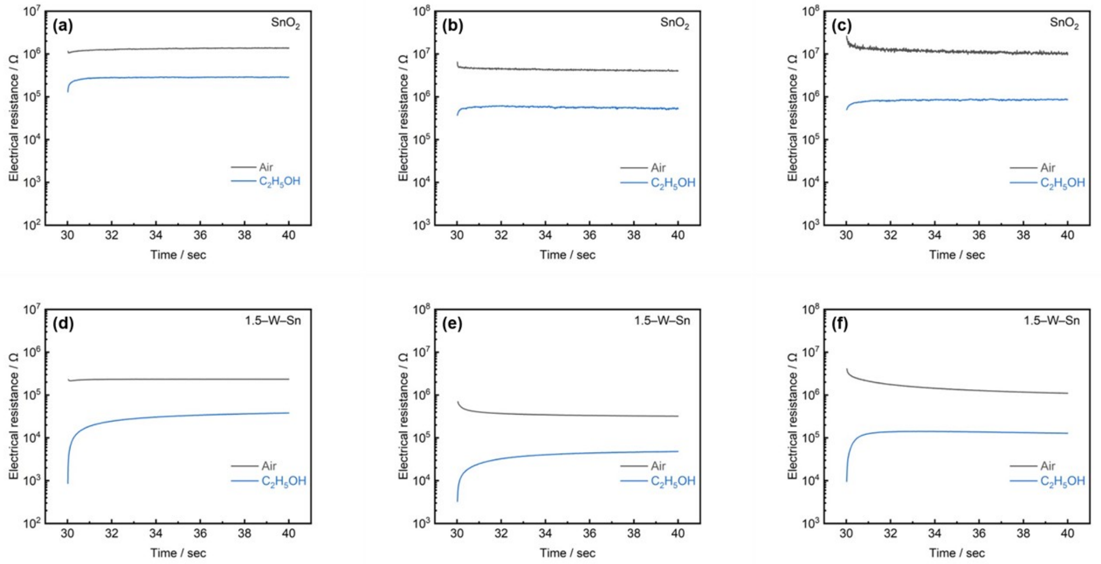

Fig. S9. The electric resistance curves in synthetic air and 10 ppm ethanol/air during a pulse heating step at 350, 300 and 250 °C under double-pulse-driven mode using (a–c) SnO_2, (d–f) 1.5–W–Sn, respectively.

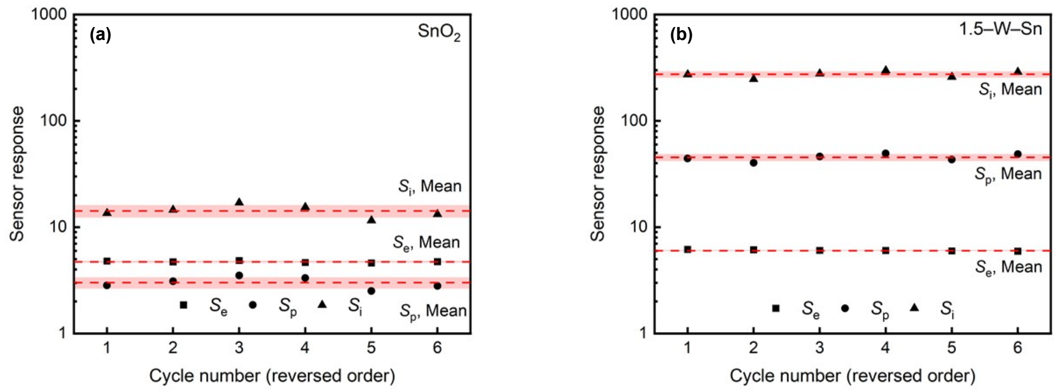

Fig. S10. Stability of the sensor response for the (a) SnO_2 and (b) 1.5–W–Sn sensors to 10 ppm ethanol at measurement temperature of 350 °C under DP mode.

Stability of the sensor responses (S_e, S_p, and S_i) for (a) neat SnO_2 and (b) 1.5–W–Sn sensors toward 10 ppm ethanol at a measurement temperature of 350 °C under DP mode was shown in Fig. S10. The data points represent the responses from 6 representative consecutive cycles. The dashed red lines indicate the mean value for each response parameter. The Relative Standard Deviation values for 1.5–W–Sn were calculated to be 1.4% (S_e), 7.5% (S_p), and 6.9% (S_i).
